# Gratifications for Social Media Use in Entrepreneurship Courses: Learners’ Perspective

**DOI:** 10.3389/fpsyg.2019.01270

**Published:** 2019-05-31

**Authors:** Yenchun Wu, Dafong Song

**Affiliations:** Graduate Institute of Global Business and Strategy, National Taiwan Normal University, Taipei, Taiwan

**Keywords:** entrepreneurship education (EE), entrepreneurship (new firms, start-ups), social media, uses and gratifications theory, Line, Facebook (FB), Wechat, entrepreneurship

## Abstract

The purpose of this study is to explore uses and gratifications on social media in entrepreneurship courses from the learners’ perspective. The respondents must have participated in government or private entrepreneurship courses and joined the online group of those courses. Respondents are not college students, but more entrepreneurs, and their multi-attribute makes the research results and explanatory more abundant. A total of 458 valid data was collected. The results of the survey revealed four gratification factors namely trust, profit, learning, and social in online entrepreneurial groups. It is also found that the structures and of the four gratification factors vary in three social media (Line, Facebook, and WeChat) and “trust” outranks other factors. Most of the entrepreneurs’ business is “networking business,” and the business unit is mostly “micro.” In terms of the trust factor, there are significant differences among the three social media. In short, the two gratification factors of trust and profit can be seen as specific gratifications for online entrepreneurial groups, especially the trust factor, which deserves more attention in the further research of online entrepreneurial courses on social media.

## Introduction

“Entrepreneurs are not afraid of more brothers.” This proverb refers to the spirit of entrepreneurs and the reason that they prefer joining groups and are particularly inclined to form a community. In recent years, social media has become a popular tool for entrepreneurs to cluster around. Numerous entrepreneurship courses have begun to use social media as a teaching aid (Menkhoff and Bengtsson, 2012; Lapolla, 2014). It can help entrepreneurial teachers improve their teaching practices to increase learner engagement and support learners’ success in individual courses, learning experiences, and teaching goals. In addition, from the perspective of the development of entrepreneurship education differs from that of general subjects. Entrepreneurship is fascinating but challenging. Because it involves uncertainty and risk, most entrepreneurship courses replace traditional teaching with mentoring, coaching and inheriting experience are more popular among learners than are the theories in books. In this context, the diverse and unique functions of social media are highly conducive to the development of entrepreneurship courses. Social media use can promote the development of learners and of the teacher-learner relationships. Emerging digital teaching technologies can facilitate interaction between learners and course content, increase learners’ motivation, enhance their entrepreneurial skills, and increase learner engagement in entrepreneurial knowledge acquisition (Wu et al., 2017). However, many entrepreneurs do not become unsuccessful in the first attempt, and thus, entrepreneurship courses can invite successful learners back to class to share their success stories. In this case, social media provides an effective learning platform; learners can find help in their entrepreneurial activities through access to numerous online resources. Based on the abovementioned points, we regarded social media as a teaching tool, particularly beneficial for entrepreneurial learning, and selected the online groups of entrepreneurial courses as the object of observation.

Encouraging teachers to use social media as a teaching strategy is worthwhile (Gruzd et al., 2018). However, for a learner-centered position, the following questions are raised: what are the reasons for learners to continue the use social media in the curriculum design? Can social media genuinely enhance the absorption and application of learners’ knowledge in the classroom? What are the factors that help, interfere with, and cause doubt when learners use this knowledge? In general, social media must be sustained, actively invested in, and used by team members to achieve its value. However, research and commentary on applying social media to entrepreneurship courses are rare. Therefore, how to effectively use the particularities of social media to attract learners to use it voluntarily and continually is worthy of further discussion.

Course groups can use social media platforms, such as Line, Facebook, and WeChat. Some groups are a course requirement for teachers and some are composed of learners themselves; some groups can be joined for free and some require a fee. We found that in terms of groups’ growth and decline, in addition to the characteristics of the social media platform, the active participation of team members is the most critical factor. Therefore, this study focused on the online groups of entrepreneurship courses to explore the experiences of learners in their curriculum and motivation to continue to use social media.

The uses and gratifications (U&G) theory emphasizes that an audience will actively select and use a particular form of media according to their own needs and then use their behaviors to meet its demands (Katz et al., 1973, 1974). Social media is highly suitable for being discussed from the perspective of the U&G theory because it is a medium that people use to attract other users, who they then use to improve and expand their own media. Furthermore, the idea that learners use social media in a targeted manner is quite consistent with the U&G theory, which considers users to be active decision-makers who seek, use, and apply media for their own purposes (Katz et al., 1973, 1974; Shao, 2009). To answer the research questions, this study explored the characteristics of social media and the motivation and gratification of learners in social media through the U&G theory.

Many studies conducted on the relationship between social media and learning have been set in schools. The research targets have mostly been teachers (Gruzd et al., 2018) and students (Menkhoff and Bengtsson, 2012; Dahlstrom et al., 2013; Lapolla, 2014; Lupton, 2014; Wohn and LaRose, 2014; Barhoumi, 2015; Gan and Wang, 2015; Imlawi et al., 2015; Hajibayova, 2017; Gan, 2018; Klobas et al., 2018). However, as far as entrepreneurship courses are concerned, apart from schools, the government and the private sector have vigorously promoted the courses in quantity and terms of types even beyond school education. The current study used government and private entrepreneurship course learners as research objects for two reasons: first, the diversity of personal attributes (e.g., age and education level) can enrich the interpretation of the research results, and second, such learners may have relatively stronger entrepreneurial motivations as well as experiences of entrepreneurship success or failure. Therefore, we attempted to answer the following research questions: how do these learners use social media in their courses? What are the purposes and gratification factors of using social media? What are the challenges and difficulties of using social media?

If teachers understand the needs of entrepreneurship learners, they can use social media correctly and more effectively to help achieve their teaching goals. Most of the relevant studies have focused on the use of one or two social media platform (Wohn and LaRose, 2014; Gan and Wang, 2015; Gan, 2018; Klobas et al., 2018), but the comparative analyses of the use of three social media platforms have been limited. Therefore, this study compared the similarities and differences of gratification factors across three social media. Thus, this study’s objectives are as follows: (1) To explore the current situation of learners using social media in entrepreneurship courses. (2) To investigate the learners’ perspective the gratification factors of using social media in entrepreneurship courses. (3) To compare the similarities and differences among the gratification factors of three social media entrepreneurship courses.

### Literature Review

#### Social Media and Learning

The contribution of social media in the classroom has generated increasing interest in such tools for assisted learning. Six reasons for teachers to use social media in their curriculum are that it (1) promotes student participation, (2) makes teachers more organized, (3) combines external resources; (4) makes students more focused on the topic being learned, (5) establishes a group for practical applications, and (6) enables resource development (Gruzd et al., 2018). Some studies from students’ perspective also exist. For example, Dahlstrom et al. (2013) surveyed 113,035 college students from 14 countries on technology perception and use in higher education. Although the students understood the value of learning on social media, they wanted to know how to apply social media more effectively in the courses they study themselves. Similarly, in a study on six courses, each course used a different type of social media differently; it found that students struggled to use social media because they felt that they did not learn enough about how to use teaching support for social media (Bennett et al., 2012). Wohn and LaRose (2014) found that the compulsive use of YouTube aided students in studying their courses; however, the negative feelings were similar to the consequences of being forced to use Facebook. This discovery may alert teachers of the risk of forced use when applying social media. However, studies on social media and learning from these perspectives are limited, particularly those related to learning; they are usually related to learning how to use social media rather than applying it to the curriculum to enhance the learning effects of the course.

Other classroom applications include those reported: Wang et al. (2012) who used Facebook as a two-course learning management system; Barhoumi (2015) and Sulisworo and Toifur (2016), who incorporated WhatsApp; and Menkhoff and Bengtsson (2012) who applied mobile phones and social media to entrepreneurship courses at a university in Singapore. These studies have shown that students were willing to use and satisfied with social media as a teaching tool and that the learning results were good.

Lapolla (2014) evaluated the use of the social media platform Pinterest in fashion design courses and found that students believed they benefited from communicating with customers through the community. This indicated that extended learning was considered an intention of using social media. In a survey conducted by Lupton (2014) the reasons respondents gave for using social media were working with other people in the class and for outside learning and extending classroom contact time. Therefore, expanding the classroom contact environment is a reason for using social media. This validates the use of social media and expands the scope of the learning environment to access resources. However, in the aforementioned literature, the research targets were still mostly teachers and students.

The use of social media in courses allows learners to experience active participation, sharing, openness, and collaboration, and simultaneously attracting them to actively engage in and even start their career (Senges et al., 2008). Furthermore, the use of social media helps promote participants’ development. This matches Vygotsky’s (1978) view, who advocated that learners’ ability to solve problems with the help of a teacher or cooperation with a more capable companion goes beyond the problem-solving ability that emerges when they are alone, he believed that the group provides an interactive situation wherein teachers provide guidance and support through the dialog to enhance the ability to expand learners’ knowledge and demonstrate different learning outcomes. Because the development of individuals is influenced by social interactions, the process of group interaction learning can also promote individual development, which makes participation and reflection another reason for using social media in the curriculum. Learners must interact with others and learn and practice new norms to actively form their own learning style.

Teachers are also crucial players in the group. The personality traits and interactions of teachers may be a reason that learners continue to participate. Imlawi et al. (2015) explored the use of social media in a study on teacher credibility and found that when teachers posted information about themselves and expressed a sense of humor, students’ learning outcomes were stimulated, which could be considered a method of teachers and students using social media to build trust and cooperation. When we interviewed respondents about the entrepreneurship curriculum, nearly everybody mentioned that trust is a crucial reason affecting their motivation to join or leave the group.

In summary, there are several reasons for learners using social media in their courses: (1) it aids learners in actually experiencing what they have learned; (2) it expands the scope of the learning environment; (3) it promotes interaction, cooperation, and learning; and (4) it highlights the personality traits of teacher and encourages their participation.

#### Uses and Gratifications Theory and Social Media

U&G theory identifies the social and psychological needs that drive individuals to use specific media, and it also explains why individuals actively choose particular media to gratify a variety of needs (Katz et al., 1974). U&G theory pays great attention to the initiative of the audience, emphasizing the voluntary and selective nature between the audience and the media, which is highly suitable for the theme of this study. In this era of continual introductions of new forms of media, when the initiative of the audience becomes a key factor in the behavior of the media, the U&G are more suitable for the overall picture of the audience’s use of media behavior. Social media may not be able to replace traditional media but it may help researchers explore the motivations, needs, and gratifications of more audiences.

Some of the studies using U&G perspective are as follows: Gan (2018) analyzed 368 Chinese college students who used the social media platforms. Sina Weibo and WeChat and confirmed that four types of gratification exist across different social media platforms: hedonic, emotional, message, and social gratification. Furthermore, the author found that the intensity of each gratification varied to various degrees in terms of the use of different social media platforms. Klobas et al. (2018) studied 807 Malaysian university students and found that entertainment motivation is more attractive to students than messages motivation.

The U&G framework fits our research because it does not assume a set of predefined gratifications factors, but rather, causes factors to be generated from the data; this led us to ask how and why such media is used as well as how each type of media serves each user. Thus, corroborating to our research objectives, we selected respondents who used entrepreneurial course groups on Line, WeChat, or Facebook.

## Materials and Methods

### Questionnaire Design and Identification

Our questionnaire was aimed at understanding the current situation of learners who use social media in entrepreneurship courses as well as at exploring the gratifications factors of using social media in entrepreneurship courses from the learners’ perspective. First, we interviewed five well-known entrepreneurship teachers and 12 active members of entrepreneurship course groups and held two focus group discussions. We developed questions relevant to our research objectives, and then, according to the interview content and literature review, we incorporated them into the initial questionnaire. This questionnaire was evaluated for expert validity by six senior entrepreneurship teachers, and corrections were made to form the pretest questionnaire.

Initially, the questionnaire was based on the four directions in the literature: (1) the learner actually experiencing what he or she has learned, (2) expanding the scope of the learning environment, (3) promoting learning through group interaction and cooperation, and (4) encouraging the teacher’s traits and the participation levels. However, after the expert interviews and focus group discussions, the traits of the teacher were removed because groups formed by learners did not necessarily have a teacher and some entrepreneurship courses online are comprised of only learners. The pretest questionnaire consisted of two parts. The first part concerned the feeling of participating in the group, and the content included the five aspects of trust, profit, learning, happiness, and interaction. In total, the first part comprised 21 questions, all of which were measured using a five-point Likert scale, ranging from 1 (strongly disagree) to 5 (strongly agree). The second part concerned personal use and basic information. To obtain the degree of discrimination for the pretest questionnaire, we conducted item analysis. As presented in Table 1, the composite reliability (CR) of 21 items reached significance, indicating that each item had a good degree of discrimination. Furthermore, the total correlation coefficient (CC) was > 0.4, which also reached significance; thus, all 21 items were retained.

**Table 1:** After item analysis and factor analysis the removed questions, the new facets, and the new question number.

Original facets and questions		CC	CR	Removed	New facet	New no.
Trust	(1) I believe that most of the information provided by this group is reliable.	0.66^***^	9.92^***^		**Trust**	1
	(2) I think the teacher in the courses can be trusted.	0.69^***^	10.09^***^		**Trust**	2
	(3) I think most of the members of this group are trustworthy.	0.67^***^	9.61^***^		**Trust**	3
	(4) Even if the exchange of information in a group is risky, I will still participate in the group.	0.47^***^	5.27^***^	X		
Profit	(5) Joining the group gives me a chance to make money.	0.70^***^	11.51^***^		**Profit**	4
	(6) The participation of the group expanded my network and helped me start a career.	0.78^***^	12.96^***^		**Profit**	5
	(7) The main purpose of participating in the group is to make money in order to start a business.	0.71^***^	10.89^***^		**Profit**	6
	(8) I think we can take advantage of starting a business through the community.	0.70^***^	9.87^***^	X		
	(9) Joining the group can help me think of a new idea to start a career.	0.71^***^	10.00^***^	X		
Learning	(10) Participate in the group to help me better understand the meaning of entrepreneurship or heterogeneous alliance and other concepts.	0.66^***^	9.38^***^		**Learning**	7
	(11) Joining the group can help me review what I have learned after going home.	0.75^***^	12.65^***^		**Learning**	8
	(12) Sharing or discussing in the group helps me with my studies.	0.78^***^	11.12^***^		**Learning**	9
	(13) The information published by the group members in the cluster is more relevant to the subject matter of the course.	0.72^***^	13.50^***^		**Learning**	10
Happiness	(14) When I interact with other team members, it makes me feel very happy.	0.30^***^	2.82^***^	X		
	(15) Someone in the group answered my question and made me feel warm.	0.69^***^	10.07^***^	X		
	(16) It makes me feel comfortable to express my opinions or ask questions in the group.	0.74^***^	12.61^***^		**Social**	12
	(17) When I send a message in a group, someone gives me a sense of laud when he likes it.	0.71^***^	10.23^***^		**Social**	13
Interaction	(18) Teachers or members of the team often respond to questions brought up in the group.	0.79^***^	14.36^***^		**Learning**	11
	(19) The interaction between the team members is consistent.	0.70^***^	9.45^***^		**Social**	14
	(20) I often interact with other team members.	0.70^***^	11.03^***^		**Social**	15
	(21) Groups often organize gatherings and invite team members to participate.	0.66^***^	9.80^***^		**Social**	16


*^∗∗∗^*p* < 0.000.*

Subsequently, factor analysis was performed, followed by Kaiser-Meyer-Olkin (KMO) verification and Bartlett’s test to determine whether the data was suitable for factor analysis. Next, principal component analysis was conducted to extract common factors, and through Varimax rotation the selected factors were rotated. The factor loadings of each item are required to be > 0.5.

After five rounds of factor analysis, five items were removed individually: question 4 of the trust facet, questions 8 and 9 of the profit facet, and questions 14 and 15 of the happiness facet. According to Table 2, the remaining 16 items have KMO = 0.94 > 0.9, Bartlett’s test = 2746.60, and Sig. = 0.000, which indicated that they were suitable for factor analysis, and reduced the original five facets to four. Question 18 had originally belonged to the interaction facet before, belonging to the learning facet, and questions 16 and 17 had originally belonged to the happiness facet before, belonging to the interaction facet. The happiness and interaction facets were combined to form one facet, which was called “social” (see Table 1). According to Table 2, the eigenvalues of all four facets greater than 1: trust (2.60), profit (2.42), learning (3.11), and social (3.72). The variance explained was 16.26% for trust, 15.13% for profit, 23.22% for social, and 19.46% for learning, and the highest explanatory power was for social. The total variance explained was 74.07 > 50.0%, indicating that the first part of the questionnaire had good validity and explanatory power. The first part of the new questionnaire concerned the feeling of participating in the group. It was divided into four facets and 16 items as follows: trust comprised three items, profit comprised three items, learning comprised five items, and social comprised five items. Next, reliability analysis was performed, the overall questionnaire Cronbach’s α = 0.94, and the Cronbach’s α of each facet: trust (0.84), profit (0.84), learning (0.89), and social (0.88), all of which were > 0.7 (see Table 2), indicating that the overall questionnaire and each facet had stable internal consistency. Therefore, the questionnaire was valid as a tool for follow-up research. We continued to use this questionnaire to investigate and analyze entrepreneurship course groups on the following three social media platforms: Line, Facebook, and WeChat.

**Table 2:** Factors extracted for pre-test questionnaire.

	Pre-test (=173)			
	
I think/I feel use Line/Facebook/WeChat	F1	F2	F3	F4
**F1: Trust**				
Message is reliable	0.839			
Teacher can be trusted	0.763			
Members are trustworthy	0.699			
**F2: Profit**				
Have a chance to make money		0.784		
Expanded my network		0.594		
In order to start a business		0.755		
**F3: Learning**				
Understand the meaning of entrepreneurship			0.596	
Can review what I have learned			0.757	
Help me with my studies			0.715	
Relevant to the subject matter of the course			0.701	
It often respond to questions brought up			0.664	
**F4: Social**				
It makes me feel comfortable				0.678
Someone gives me a sense when he likes it				0.709
Interaction between the members is consistent				0.770
I often interact with members				0.761
It often organize physical gatherings				0.736
**Eigenvalue**	2.60	2.42	3.11	3.72
**Variance explained (%)**	16.26	15.13	19.46	23.22
**Total variance explained (%)**	74.01			
**Cronbach’s α**	0.84	0.84	0.89	0.88
**Total Cronbach’s α**	0.94			
**KMO**	0.94			
**Bartlett’s test**	1852.18 Sig. = 0.000			


### Data Collection

The respondents were required to have taken an entrepreneurship course and participated in its online group on Line, Facebook, or WeChat. The target audience was entrepreneurship groups that we had participated in and were familiar with or entrepreneurial groups recommended by senior entrepreneurial teachers. Some online groups are designed by teachers for their curriculum, whereas some are comprised of the learners themselves. Using a group message notification, we sent a message to respondents containing a link to the questionnaire on an online survey website. In addition to the online survey system, we participated in the physical group of the entrepreneurship courses to interview learners and collect questionnaires. We not only employed group messaging but also one-to-one messaging for anonymous answers. Two online questionnaire systems were employed: Google Questionnaire (in Traditional Chinese), and Tencent Questionnaire (in Simplified Chinese), which were posted on the entrepreneurship groups on Line, Facebook, and WeChat. In total, 42 entrepreneurship course groups were interviewed (Line = 21, Facebook = 16, and WeChat = 5) and the number of members in each group was between 23 and 463. In addition, a questionnaire setting mechanism was applied to avoid missed items and repeated responses by the respondents.

## Results

### Respondent Demographics

The respondents’ demographics are shown in Table 3. In total, 173 questionnaires were collected from the pretest subjects, and 458 questionnaires were collected from the formal test subjects (Line = 189, Facebook = 142, and WeChat = 127). The ratio of men to women is roughly equal. Regarding age, the majority of WeChat users were aged 21–30 years, whereas the users of the other groups were mostly aged 31–50 years. Most respondents had a university-level education. The proportion of respondents who used the group because it was a part of their teacher’s curriculum design was 42.8–51.2%; that of respondents who were required to pay for the group was 33.3–51.2%; and that of respondents who used the group several times a day was 34.4–66.9%. Furthermore, more than half of the respondents had already started their own business (62.5–91.0%). As shown in Table 4, the business units are mostly “micro” (employing fewer than five people) and most of the businesses are “networking business.” Other business types included dining, cram schools, investment, and beauty and etc. As for the other items, many types existed, indicating that the categories of entrepreneurship are quite diverse.

**Table 3:** Respondent demographics.

	Pre-test (*n* = 173)	Line (*n* = 189)	Facebook (*n* = 142)	WeChat (*n* = 127)
				
Measure and items	Frequency (%)	Frequency (%)	Frequency (%)	Frequency (%)
**Gender**				
Male	88 (50.9)	101 (53.4)	62 (43.7)	61 (48.0)
Female	85 (49.1)	88 (46.6)	80 (56.3)	66 (52.0)
**Age**				
21–30	34 (19.6)	21 (11.1)	21 (14.8)	62 (48.8)
31–40	51 (29.5)	58 (30.7)	47 (33.1)	25 (19.7)
41–50	53 (30.6)	64 (33.9)	47 (33.1)	23 (18.1)
51–60	26 (15.0)	40 (21.2)	21 (14.8)	12 (9.5)
Over 61	9 (5.2)	6 (3.1)	6 (4.2)	5 (3.9)
**Education**				
Under high school	11 (6.4)	5 (2.6)	6 (4.2)	9 (7.1)
High school	32 (18.5)	19 (10.1)	18 (12.7)	21 (16.5)
University	90 (52.0)	98 (51.9)	78 (54.9)	76 (59.8)
Graduate school	40 (23.1)	67 (35.4)	40 (28.2)	21 (16.5)
**Type**				
Teacher’s design	74 (42.8)	85 (45.0)	65 (45.8)	65 (51.2)
Learners’ self-contained	99 (57.2)	104 (55.0)	77 (54.2)	62 (48.8)
**Need to pay**				
Yes	67 (38.7)	63 (33.3)	56 (39.4)	65 (51.2)
No	106 (61.3)	126 (66.7)	86 (60.6)	62 (48.8)
**How long of use**				
Less than 6 months	49 (28.3)	69 (36.5)	29 (20.4)	52 (40.9)
6–12 months	51 (29.5)	82 (43.4)	44 (31.0)	49 (38.6)
1–2 years	33 (19.1)	31 (16.4)	36 (25.4)	9 (7.1)
More than 2 years	40 (23.1)	7 (3.7)	33 (23.2)	17 (13.4)
**How often**				
Once a week or longer	20 (11.6)	9 (4.8)	6 (4.2)	1 (0.8)
Several times a week	27 (15.6)	52 (27.5)	18 (12.7)	14 (11.0)
Once a day	26 (15.0)	60 (31.7)	14 (9.9)	38 (29.9)
Several times a day	76 (43.9)	65 (34.4)	95 (66.9)	65 (51.25)
All the day	24 (13.9)	3 (1.6)	9 (6.3)	9 (7.1)
**Place of residence**				
Taiwan	149 (86.1)	187 (98.9)	131 (92.3)	6 (4.7)
China	22 (12.7)	2 (1.1)	5 (3.5)	118 (92.9)
Others	2 (1.2)		6 (4.2)	3 (2.4)


**Table 4:** Industry information of entrepreneurs.

	Pre-test (*n* = 134)	Line (*n* = 172)	Facebook (*n* = 113)	WeChat (*n* = 79)
				
Measure	Frequency (%)	Frequency (%)	Frequency (%)	Frequency (%)
**Have you started your business**				
Yes	134 (77.5)	172 (91.0)	113 (79.6)	79 (62.5)
No	39 (22.5)	17 (9.0)	29 (20.4)	48 (37.8)
**Size of the business**				
Micro	111 (82.8)	135 (78.5)	100 (88.5)	71 (89.9)
Small	20 (14.9)	37 (21.5)	12 (10.6)	8 (10.1)
Medium	3 (2.2)	0	1 (0.9)	0
**What business do you run**				
Networking	41 (30.6)	33 (19.2)	44 (38.9)	30 (38.0)
Dining/baking	20 (14.9)	12 (7.0)	15 (13.3)	11 (13.9)
Investment	16 (11.9)	10 (5.8)	7 (6.2)	6 (7.6)
Cram classes	14 (10.5)	16 (9.3)	13 (11.5)	10 (12.7)
Beauty	8 (6.0)	13 (7.6)	12 (10.6)	8 (10.1)
Others	35 (26.1)	88 (51.2)	22 (19.5)	14 (17.7)


### Gratifications for the Use of Three Social Media

As shown in Table 5, the factors extracted from Line have KMO = 0.91 > 0.9, Bartlett’s test = 2025.72, Sig. = 0.000, which indicated that they were suitable for factor analysis. The eigenvalues of all four facets were greater than 1: trust = 2.48, profit = 2.57, learning = 3.19, social = 3.58. The total variance explained is 73.81%, the interpretation of social is the highest (22.36%). Regarding the reliability analysis, the overall questionnaire Cronbach’s α = 0.93, trust Cronbach’s α = 0.80, profit Cronbach’s α = 0.82, learning Cronbach’s α = 0.89, social Cronbach’s α = 0.90, all of which were > 0.7, indicating that the overall questionnaire and each facet have stable internal consistency and the gratification factors have good validity and explanatory power.

**Table 5:** Factors extracted and reliability for three social media.

	Line (*n* = 189)				Facebook (*n* = 142)				WeChat (*n* = 127)			
			
I think/I feel	F1	F2	F3	F4	F1	F2	F3	F4	F1	F2	F3	F4
**F1: Trust**												
Message is reliable	0.825				0.861				0.782			
Teacher can be trusted	0.742				0.714				0.769			
Members are trustworthy	0.721				0.788				0.741			
**F2: Profit**												
Have a chance to make money		0.735				0.733				0.797		
Expanded my network		0.773				0.606				0.596		
In order to start a business		0.838				0.798				0.753		
**F3: Learning**												
Understand the meaning of entrepreneurship			0.635				0.754				0.500	
Can review what I have learned			0.687				0.619				0.721	
Help me with my studies			0.763				0.656				0.703	
Relevant to the subject matter of the course			0.733				0.624				0.780	
Often respond to questions brought up			0.648				0.552				0.692	
**F4: Social**												
It makes me feel comfortable				0.621				0.668				0.652
Someone gives me a sense when he likes it				0.609				0.772				0.530
Interaction between the members is consistent				0.775				0.757				0.716
I often interact with members				0.854				0.673				0.787
Often organize physical gatherings				0.755				0.771				0.781
**Eigenvalue**	2.48	2.57	3.19	3.58	2.89	2.32	2.86	3.76	3.06	2.20	2.90	3.04
**Variance explained (%)**	15.47	16.07	19.91	22.36	18.07	14.5	17.84	23.49	19.11	13.76	18.11	19.03
**Total variance explained (%)**	73.81				73.9				70.01			
**Cronbach’s α**	0.80	0.82	0.89	0.89	0.85	0.84	0.89	0.88	0.83	0.85	0.85	0.83
**Total Cronbach’s α**	0.93				0.94				0.93			
**KMO**	0.91				0.93				0.92			
**Bartlett’s test**	2025.72 Sig. = 0.000				1561.80 Sig. = 0.000				1126.88 Sig. = 0.000			


The factors extracted from Facebook have KMO = 0.93 > 0.9, Bartlett’s test = 1561.79, Sig. = 0.000, which indicated that they were suitable for factor analysis. The eigenvalues of all four facets are greater than 1: trust = 2.89, profit = 2.32, learning = 2.86, social = 3.76, the total variance explaining 73.90%, the interpretation of social is the highest (23.49%). Reliability analysis, the overall questionnaire Cronbach’s α = 0.94, trust Cronbach’s α = 0.85, profit Cronbach’s α = 0.84, learning Cronbach’s α = 0.89, social Cronbach’s α = 0.88, all of which were > 0.7, indicating that the overall questionnaire and each facet have stable internal consistency and the gratification factors have good validity and explanatory power.

The factors extracted from WeChat have KMO = 0.92 > 0.9, Bartlett’s test = 1126.88, Sig. = 0.000, which indicated that they were suitable for factor analysis. The eigenvalues of all four facets are greater than 1: trust = 3.06, profit = 2.20, learning = 2.90, social = 3.04, the total variance explaining 70.01%, the interpretation of trust is the highest (19.11%). Reliability analysis, the overall questionnaire Cronbach’s α = 0.93, trust Cronbach’s α = 0.83, profit Cronbach’s α = 0.85, learning Cronbach’s α = 0.85, social Cronbach’s α = 0.83, all of which were > 0.7, indicating that the overall questionnaire and each facet have stable internal consistency and the gratification factors have good validity and explanatory power. This outcome has the similar result as the study by Gan and Wang (2015) and Gan (2018), the structure of the gratification factor is different in different social media. It shows that although users can get the same gratification from different social media, how get the gratification may vary.

### Comparative Analysis of the Gratifications on Three Social Media

Table 6 presents the means and rankings of the four factors across the three social media platforms. The gratification ranking was as follows: trust, learning, profit, and social. Trust ranked first across all social media platforms. Subsequently, we conducted an analysis of the variance. The results of Levene’s test indicated that two facets exhibited inhomogeneity of variance (see Table 7); therefore, we referred to the Brown-Forsythe and Welch statistics to determine the average (robust tests of equality of means). Both types of statistics followed an *F* distribution, and thus the homogeneity assumption was not required. The verification results were significant (see Table 8), and therefore, *post hoc* analysis was continued. Because the number of groups exceeded 50, we used Games–Howell tests for *post hoc* comparisons. The results are shown in Table 9. Regarding the trust factor, the difference between on three social media is significant (WeChat > Facebook > Line). Regarding the profit factor, the difference between WeChat and Line is significant (WeChat > Line) and that between Facebook and Line was significant (Facebook > Line); however, the difference between WeChat and Facebook is not significant. Regarding the learning factor, the difference between WeChat and Line is significant (WeChat > Line) and that between Facebook and Line is significant (Facebook > Line); however, the difference between WeChat and Facebook is not significant. Finally, regarding the social factor, the difference between WeChat and Line is significant (WeChat > Line) and that between Facebook and Line is significant (Facebook > Line); however, the difference between WeChat and Facebook is not significant.

**Table 6:** Mean and ranking of o four factors for three social media.

	Line (*n* = 189)			Facebook (*n* = 142)			WeChat (*n* = 127)		
			
Factor	Mean	*SD*	Ranking	Mean	*SD*	Ranking	Mean	*SD*	Ranking
Trust	3.67	0.82	1	3.90	0.85	1	4.14	0.76	1
Profit	3.26	1.03	3	3.77	0.97	3	3.74	0.94	3
Learning	3.52	0.96	2	3.83	0.86	2	3.91	0.76	2
Social	3.07	1.02	4	3.67	0.86	4	3.73	0.81	4


**Table 7:** Test of homogeneity of variances.

Factor	Levene statistic	df1	df2	Sig.
Trust	1.90	2	455	0.151
Profit	0.54	2	455	0.581
Learning	5.27	2	455	0.005
Social	4.18	2	455	0.016


**Table 8:** Robust tests of equality of means.

Factor		Statistic^a^	df1	df2	Sig.
Trust	Welch	13.62	2	287.1	0.000
	Brown-Forsythe	12.93	2	431.5	0.000
Profit	Welch	13.24	2	289.6	0.000
	Brown-Forsythe	13.86	2	439.6	0.000
Learning	Welch	8.75	2	295.3	0.000
	Brown-Forsythe	9.33	2	450.3	0.000
Social	Welch	24.49	2	295.8	0.000
	Brown-Forsythe	27.42	2	452.8	0.000


*^*a*^Asymptotically *F* distributed.*

**Table 9:** Mean comparison among three social media.

Factor			Mean difference	Sig.	*Post hoc* tests (Games–Howell)
Trust	Line	WeChat	0.47^*^	0.000	WeChat > Facebook > Line
		Facebook	-0.23^*^	0.038	
	WeChat	Line	0.47^*^	0.000	
		Facebook	0.24^*^	0.038	
	Facebook	Line	0.23^*^	0.038	
		WeChat	-0.24^*^	0.038	
Profit	Line	WeChat	-0.48^*^	0.000	WeChat > Line Facebook > Line WeChat = Facebook
		Facebook	-0.50^*^	0.000	
	WeChat	Line	0.48^*^	0.000	
		Facebook	-0.03	0.975	
	Facebook	Line	0.50^*^	0.000	
		WeChat	0.03	0.975	
Learning	Line	WeChat	-0.39^*^	0.000	WeChat > Line Facebook > Line WeChat = Facebook
		Facebook	-0.31^*^	0.006	
	WeChat	Line	0.39^*^	0.000	
		Facebook	0.08	0.704	
	Facebook	Line	0.31^*^	0.006	
		WeChat	-0.08	0.704	
Social	Line	WeChat	-0.66^*^	0.000	WeChat > Line Facebook > Line WeChat = Facebook
		Facebook	-0.60^*^	0.000	
	WeChat	Line	0.66^*^	0.000	
		Facebook	0.06	0.841	
	Facebook	Line	0.60^*^	0.000	
		WeChat	-0.06	0.841	


*^∗^*p* < 0.05, *N* = 458.*

### Confirmatory Factor Analysis

**FIGURE 1:**
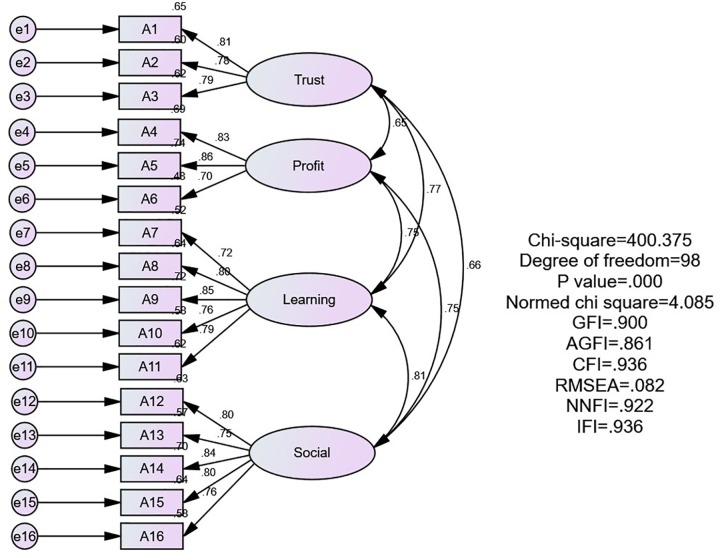
Confirmatory factor analysis (CFA) for the four dimension.

As shown in Figure 1, confirmatory factor analysis (CFA) of the 458 formal respondents revealed the following results: χ^2^(98, *N* = 458) = 400.375, *P* = 0.001, χ^2^/df = 4.058 < 5, GFI = 0.90 > 0.9, AGFI = 0.86 > 0.8, CFI = 0.94 > 0.9, NNFI = 0.92 > 0.9, IFI = 0.94 > 0.9, RMSEA = 0.08 < 0.1, indicating that the model’s fit could be accepted (Bagozzi and Yi, 1988). As shown in Table 10, the factor loading of 16 items are all > 0.7, indicating good convergent validity. The CR of the four factors is as follows: trust = 0.84, profit = 0.87, learning = 0.88, and social = 0.90. All are > 0.6, indicating that the measurement is stable and have good reliability. Average variance extracted (AVE) is as follows: trust = 0.63, profit = 0.68, learning = 0.60, and social = 0.64. All are > 0.5, indicating that the convergent validity of the potential variable is ideal and have good operationalization. IF the AVE of each construct is greater than the square of the correlation coefficient (CC) associated with other constructs, which verified the discriminant validity of the constructs (Fornell and Larcker, 1981). The results as shown in Table 11: between trust and profit, *r* = 0.52, *r*^2^ = 0.27. trust AVE = 0.63 > 0.27, profit AVE = 0.68 > 0.27, all of which indicate good discriminant validity between trust and profit. By analogy, good distinction validity existed between trust and learning, trust and social, profit and learning, and profit and social. Only between learning and social was the AVE not greater than the *r*^2^ of the CC associated with other constructs, as shown in Table 11. Thus, the CFA of the measurement model could be accepted and both the convergent and discriminant validity are appropriate and feasible.

**Table 10:** Factor loading of items, composite reliability, and average variance extracted.

Factor	Factor loading of items					CR	AVE	Cronbach’s α
Trust	0.81	0.78	0.79			0.84	0.63	0.83
Profit	0.83	0.86	0.70			0.87	0.68	0.84
Learning	0.72	0.80	0.85	0.76	0.79	0.88	0.60	0.89
Social	0.80	0.75	0.84	0.80	0.76	0.90	0.64	0.89


*Total Cronbach’s α = 0.94.*

**Table 11:** Correlation coefficient and average variance extraction between factors.

Factor	Trust	Profit	Learning	Social
Trust	0.63 (AVE)	0.43 (*r*^2^)	0.59 (*r*^2^)	0.63 (*r*^2^)
Profit	0.65	0.68 (AVE)	0.43 (*r*^2^)	0.56 (*r*^2^)
Learning	0.77	0.75	0.60 (AVE)	0.65 (*r*^2^)
Social	0.66	0.75	0.81	0.64 (AVE)


## Discussion and Conclusion

### Discussion and Implications

To save space, the results of the in-depth interviews are discussed together. This study found learners asked questions about the course on social media in order to get a response from the teacher or classmate. In addition, because most of the learners were entrepreneurs, the groups usually provide them with advertising time for marketing their products or ideas to the group. Since the learners are classmates, trust between them is high and the chances of the transactions are relatively great. If learners continue to participate in an advanced course, they can join the associated advanced group without interrupting their learning and continuing to use the group.

We also found that trust, profit, learning, and social are the four gratification factors for learners participating in online groups. These four factors are in line with the U&G theory’s active users in a framework. Trust and profit can be regarded as specific gratification factors, and learning and social are general gratification factors. The participants in the in-depth interviews also agreed that participating in course groups could enhance the absorption and application of classroom knowledge; for example, by reviewing what was learned, group discussions and sharing can assist them in learning. The reasons for learners being willing to continue to use the groups were strengthened learning, the ability to receive information, social and emotional gratifications, and the opportunities to make money and profit. For entrepreneurship course group members, making money is the greatest reward. If learners are allowed more opportunities to make money through their group, this will increase their gratification with the group and they will continue to participate.

As for the challenges and difficulties that learners encounter in the group, the general disturbances and doubts about using course groups were as follows: members’ questions are often repeated or too simple, and the answers may not be relevant or posted unconfirmed messages.

Besides that, we found that the entrepreneurs across the three social media platforms mostly operated networking businesses. In addition to acquiring the knowledge and skills of entrepreneurship, the reasons for their participation in entrepreneurship courses and groups were to promote their products, particularly through the power of social media, to increase their performance. Almost all operators were small storefronts or individual studios with fewer than five people. This coincides with the survey findings, and the entrepreneurs’ businesses were mostly micro business. Devanatha and Saha (2018) used case studies to learn about the success of two female entrepreneurs by using social media as an entrepreneurial tool. Their study considered social media not only low-cost and low-risk but also accessible to a wide range of target consumers, and thus, an effective entrepreneurial tool.

From the in-depth interviews in the first phase of this study, we knew that some learners started their own business because of unemployment, especially young learners. This is due to the fact that the occupation is a social identity, and young people need such recognition (Formica et al., 2017), coupled with their willingness to accept the challenge of starting a business, they participate in entrepreneurship courses and through learning, to realize the dream of entrepreneurship. However, modern social and economic environments increase occupational mobility and work transitions are more frequent (Santisi et al., 2018), so the learners may experience the less linear career pathways (Formica et al., 2017; Santisi et al., 2018). Therefore, the design of entrepreneurship courses needs to consider this point of view.

On the other hand, throughout life, knowledge, and experience, the individual’s uniqueness and subjectivity are ultimately involved (Mannino and Caronia, 2017; Mannino et al., 2017), so a flexible learning model is important, for learners to get what they need, and learn to adjust and adapt for the living environment, therefor, teaching and training should consider individual differences and special educational needs (Mannino and Faraci, 2017). Entrepreneurship courses are no exception, whether it’s a physical course or on social media.

### Contributions

The major contribution of our study is to identify four gratification factors, which are key incentives for applying social media in entrepreneurship courses. In addition, the influence of these factors is fluctuation of which depends on the variety of social media. Other contributions are as follows: first, this study extended the perspective of the entrepreneurship curriculum from universities to the government and private sector. The respondents are no longer college students, but more entrepreneurs (more than 60% of the respondents of the questionnaire survey are already business owners). The multiple attributes of the respondents enriched the research results. Our findings included the current state of social media use by learners of entrepreneurship courses, the four gratification factors of social media use in the entrepreneurship curriculum, especially the two factors of trust and profit, which has been less considered in prior studies. And a comparison of the gratification factors across the three social media platforms, the U&G theory was adopted to reveal the commonalities and differences among gratifications to use different social media. These valuable findings as above deepen our understanding of user behavior in social media. Second, the rich results and collected data can be used as materials for follow-up research. For example, the four extracted gratification factors (trust, profit, learning, and social) have good validity, reliability, and explanatory power in terms of measurement application, and the revised overall questionnaire and each facet have stable internal consistency, thus, the questionnaire could be used as a tool for subsequent research. Moreover, we can establish a structural equation model, investigate the relationship between the gratification factors and the continual use of the course group. In summary, our study can supplement the lack of information in the relevant literature.

In practice, the findings will not only help teachers to correctly and effectively use social media to achieve their teaching goals but also provide a reference and examples of applications to research and development designers in social media. For instance, social media platforms should not only be designed according to users’ general gratification factors but also unique features should be designed to meet users’ specific gratification factors, such as trust and profit, and there should be more cooperation with the entrepreneurship course group in terms of content creation. In summary, our study can supplement the lack of information in the relevant literature.

### Limitations and Suggestions for Future Research

This study possesses several limitations as follows: first, our method of the study used static data to explain dynamic process; such as the main gratification factor trust is a dynamic process (Dai et al., 2018), and from the perspective of dynamic psychology, subjective perception of time has a major impact on human behavior and choice (Mannino and Caronia, 2017; Mannino et al., 2017), therefore, longitudinal research is inevitable in the future. Second, we inferred the results with small sample size, in the future, we must increase the number of respondents, particularly in the area of Mainland China. Third, the impact of social expectations and cultural biases (Granieri et al., 2017) was ignored in the questionnaire for this study and can be included in the future. Fourth, we used the U&G framework to find different gratification factors, which also suggested that while traditional dimensions in the framework have been widely used to study the use of various media, they may not be sufficient to explain the use of new social media (Liu et al., 2016), future research can integrate other theories. Fifth, this research employed quantitative research after a pre-quality study, however, we did not conduct further in-depth interviews, follow-up studies can employ further in-depth interviews after quantification to answer the questions more clearly, even found other gratification factors and motivations.

And we can conduct surveys and analyses on a certain type of entrepreneurial groups, such as beauty enterprise groups or dining groups, and compare them with each other. Subsequent research can include a more in-depth discussion and analysis based on a certain factor, such as trust which is the foundation of all communication, but how can we psychologically understand trust behaviors in social media? In the future, we can analyze and discuss the trust factor in more detail. In addition, we can increase the comparison to other platforms such as YouTube. Furthermore, future studies can be designed to allow respondents who use two social media platforms simultaneously to compare gratification factors between groups. We can also view from other perspectives, such as self-regulation, personality differences, maladaptive personality traits, health and well-being, cognition and behavior, entrepreneurial leadership, and career perspective (Gorgievski and Stephan, 2016; Gervasi et al., 2017; Granieri et al., 2017; Mannino and Faraci, 2017) to investigate the learner’ activities and actions of entrepreneurship course on social media, Future research can dive into a deeper understanding of entrepreneurs’ learning behavior and their gratifications on social media.

## Ethics Statement

Ethics approval for this research was not required as per institutional and national guidelines. 636 Consent from all research participants was obtained by virtue of survey completion.

## Author Contributions

YW took charge in developing the concept, assisting in data collection. DS designed the research, performed data analysis, and wrote this manuscript.

## Conflict of Interest Statement

The authors declare that the research was conducted in the absence of any commercial or financial relationships that could be construed as a potential conflict of interest.
